# Immune cells-derived exosomes function as a double-edged sword: role in disease progression and their therapeutic applications

**DOI:** 10.1186/s40364-022-00374-4

**Published:** 2022-05-12

**Authors:** Ali Hazrati, Sara Soudi, Kosar Malekpour, Mohammad Mahmoudi, Arezou Rahimi, Seyed Mahmoud Hashemi, Rajender S. Varma

**Affiliations:** 1 Department of Immunology, Faculty of Medical Sciences, Tarbiat Modares University, Tehran, Iran; 2 Department of Immunology, School of Medicine, Iran University of Medical Sciences, Tehran, Iran; 3 Department of Immunology, School of Medicine, Shahid Beheshti University of Medical Sciences, Tehran, Iran; 4 Regional Centre of Advanced Technologies and Materials, Czech Advanced Technology and Research Institute, Palacký University in Olomouc, Šlechtitelů 27, 783 71 Olomouc, Czech Republic

**Keywords:** Exosome, Immune cell, Pathogenesis, Therapeutic application, IEXs, Cellular medicine

## Abstract

Exosomes, ranging in size from 30 to 150 nm as identified initially via electron microscopy in 1946, are one of the extracellular vesicles (EVs) produced by many cells and have been the subject of many studies; initially, they were considered as cell wastes with the belief that cells produced exosomes to maintain homeostasis. Nowadays, it has been found that EVs secreted by different cells play a vital role in cellular communication and are usually secreted in both physiological and pathological conditions. Due to the presence of different markers and ligands on the surface of exosomes, they have paracrine, endocrine and autocrine effects in some cases. Immune cells, like other cells, can secrete exosomes that interact with surrounding cells via these vesicles. Immune system cells-derived exosomes (IEXs) induce different responses, such as increasing and decreasing the transcription of various genes and regulating cytokine production. This review deliberate the function of innate and acquired immune cells derived exosomes, their role in the pathogenesis of immune diseases, and their therapeutic appliances.

## Introduction

Exosomes are produced from various cells like the immune cells and secreted into the extracellular environment. They are generally present in mammalian peripheral blood, urine, human saliva, semen, breast milk, bronchoalveolar fluid, cerebrospinal fluid, and amniotic fluid [1, 2]. Exosomes produced from various cells have similarities and differences in size, composition, function, and surface markers [3].

Exosomes can increase the level of biomimetism by simulating what is happening in nature between cells. Although extracellular vesicles (EVs) are released from different cells that include both microvesicles (MVs) and exosomes, only exosomes have the optimal size that may be considered suitable for potential therapeutic applications [4]. It is proposed that exosomes released by normal cells always positively affect their target cells. In contrast, those released by cells under pathological conditions, such as tumors and hyperactivated immune cells, may exert dangerous, adverse, and unknown effects [4].

**Fig. 1:**
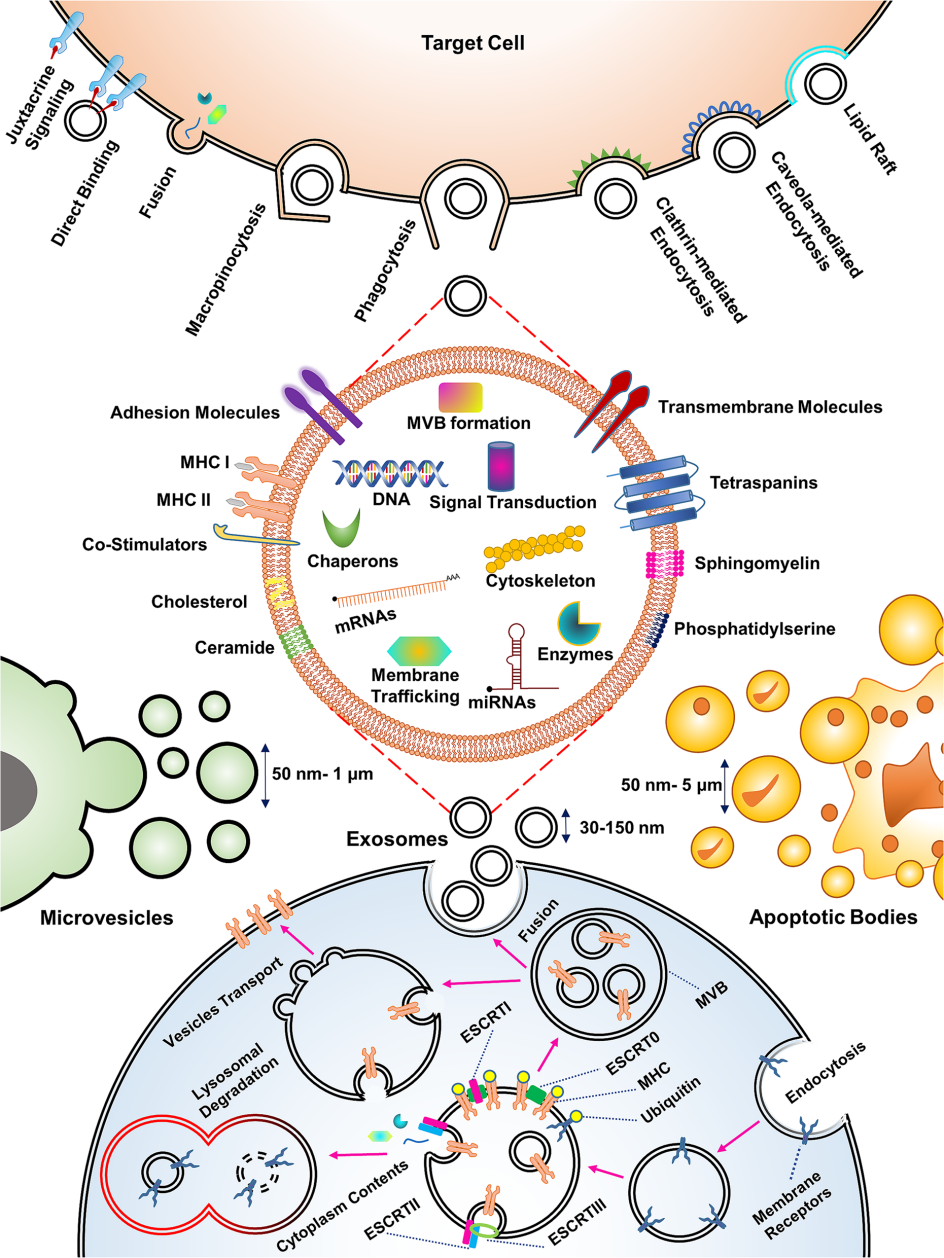
Exosome’s contents, its formation, and its capture by the target cell are shown. Interestingly, although many exosomes do not enter the phagocytic pathway and degradation, some of them are release to the cytoplasm and are destroyed by lysosomal degradation. As shown in the figure, the exosomes bind and integrate by target cell in different ways

IEXs have a broad spectrum of functions in the immune system, including expression of several types of genes, modulation of the immune system, antigen presentation, antitumor immunity, and suppressing the immune system, besides playing a critical role in the pathogenesis of certain immune-related diseases such as asthma [5] and cardiovascular disease [6]. Exosomes can be isolated using ultracentrifugation, differential centrifugation, filtration, chromatography, immunoaffinity, and deploying kits [7]. It has been shown that the dendritic cell-derived exosomes (DEX) can enhance antitumor immunity and activate specific T cells to combat against tumor cells [8]. Using methods to upregulate and downregulate exosome production by immune cells is a new way to regulate immunity against tumor and infected cells and immune reaction in some autoimmune and allergic diseases [9, 10]. Also, tumor cells secrete exosomes into their microenvironment and suppress the immune responses in this region [11]. Furthermore, inhibition of tumor cells exosome production may reduce tumor immune suppression as well as their other functions, such as angiogenesis [12]. The analysis of exosome contents leads to their characterization and determines biological applications [13]; the content of exosomes can vary depending on the origin’s cell condition, such as the stage of maturation and cell type. Exosome content is composed of three groups of macromolecules, comprising proteins, lipids, and nucleic acids [14, 15]. Exosomes’ internal compounds are divided into two groups, the first group being common for all exosomes [16]. In contrast, the second group comprises specific substances that characterize the properties and functions of exosomes [17]. Biogenesis of exosomes is initiated by inward budding of the late endosomal membrane to form multivesicular bodies (MVBs). Intraluminal vesicles (ILVs) are formed during this process in the lumen of late endosomes [18]. As shown in Fig. 1, exosome formation in different cells occurs through two pathways, including endosomal sorting complex required for transport (ESCRT) dependent and independent pathways [19, 20]. After the formation of exosomes in the late endosomes and their conversion to MVBs, they move toward the cell membrane and, after integration, exosomes release out of the cell. The cargos carried by the exosomes are transmitted to the target cell in two ways [21].

In some cases, exosome binding is sufficient to exert modifications on the target cell, but in other cases, the exosome must be internalized in addition to the binding. The initiation of exosome interaction with the target cell requires the interaction of two high-affinity surface molecules [22]. This interaction is mediated by a wide range of cell surface receptors such as integrins, cadherins, and tetraspanin [23]. Cargo transfer by exosomes to target cells modifies their functions and is performed in various ways, including soluble signaling, juxtacrine signaling, fusion, phagocytosis, macropinocytosis, receptor-mediated endocytosis, and lipid-mediated endocytosis [23, 24]. Figure 1 summarizes the biogenesis, composition, and different methods of exosomes uptake by the target cell (Fig. 1).

## Immune cells-derived exosomes (IEXs)

Immune cells, like other cells, produce exosomes to reflect their properties and create an optimal microenvironment for immune cell function where they perform their function in paracrine and autocrine forms. IEXs can activate other immune cells, inhibit immune responses, and participate in the licensing phenomenon of antigen-presenting cells (APCs) [25]. To better understand, the characteristics of the exosomes of each cell involved in acquired and innate immunity are discussed separately below.

### Dendritic cells-derived exosomes (DEXs)

DCs produce exosomes with diverse properties based on the activation nature and the presence of cytokines in the environment. If DCs are activated in inflammatory conditions, they produce exosomes that activate the innate immune system through stimulating the expression of NKG2D ligands (present on the surface of NK cells) such as MIC-A, MIC-B (MHC class I polypeptide-related sequence A and B), and ULBP-1 (UL16 binding protein-1) [26]. Also, these exosomes can activate specific T cells to detect antigens in both direct and indirect pathways [27]. In the direct pathway, DEXs can express the MHCII-peptide complexes and costimulatory molecules and bind to T cell receptors. In the indirect pathway, DEXs can deliver the MHC II-peptide complex to other DCs, a process named MHC-dressing. Besides, exosomes are able to transfer antigenic peptides from activated to inactivated DCs, which increases the number of MHCII-peptide complexes on the surface of DCs to activate the T cells. The results of new studies show that exosomes can form inside microbial compounds containing phagosomes (different types of antigens) and then be released into the immune microenvironment and participate in the antigen presentation [28]. It has also been shown that DCs-derived EVs can affect T cell responses by transferring miR-155 [29].

When DCs activated in a tolerogenic environment, they produce IL-10 and express Fas ligand (FAS-L) on their surface. Exosomes produced from these cells suppress autoimmune responses and the activity of both the innate and adaptive immune cells [30]. The expression of some proteins in the activated and inactivated DEXs are the same, such as clathrin, HSC70, annexin-2, and CD9. However, the expression levels of some proteins in the activated and activated DC-derived exosomes may differ. In the activated DC-derived exosomes, the expression levels of some molecules like MHCII, costimulatory molecules such as CD80, CD86, and CD40, as well as adhesion molecules, namely ICAM-1 increases but the expression of some molecules like MFG-E8 (Milk fat globule-epidermal growth factor-8) and MHC-I decrease [31]. Due to the unique characteristics of these exosomes, they have been investigated in various studies such as cancer immunotherapy, recruitment of mesenchymal stem cells (MSCs) [32], osteogenic differentiation of MSCs [33, 34], vaccine production for autoimmune diseases, and modulating the immune responses [35].

### Macrophages-derived exosomes

Analysis based on mass spectrophotometry has revealed the presence of ~ 5100 proteins inside the macrophages-derived exosomes, and their protein content changes after activation. For example, in the lipopolysaccharide (LPS) activated macrophages, the NOD-like receptor (NLR) related proteins will increase. Activated macrophage exosomes induce inflammation in the target cell by activating the NLRP3 receptor-dependent inflammasome, TOLL-like receptors (TLR) and TNF-related signaling pathways. Also, the levels of some cytokines and chemokines such as CXCL2, IL-17, TNF-α, CCL3, and CXCL10, are significantly increased [36] and the levels of some molecules, such as mir-17, which regulate ICAM-1 negatively, decreases in exosomes produced from activated macrophages [37]. Macrophage-derived exosomes due to the presence of mir-155 reduced the proliferation and increased the inflammation in cardiac fibroblast cells. Mir-155 has two binding sites on the 3’UTR region of the SOS1 gene. The product of this gene, a major mediator of Ras activation, is involved in proliferation by activating MAPKs (mitogen-activated protein kinases) [38], which inhibits upon binding to mir-155, and reduces cell proliferation. Likewise, mir-155 binds to the SOCS1 gene, which inhibits the secretion of inflammatory cytokines, impedes their function, and enhances the production of anti-inflammatory cytokines in cardiac fibroblast cells [39]. Macrophage-derived exosomes that activated in the inflammatory conditions can induce inflammatory conditions in liver cells [36], placenta, and vascular endothelial cells [37]. Similarly, macrophage-derived exosomes can inhibit endothelial and tumour cells’ migration by inhibiting the expression of β1 integrins [40].

Exosomes produced from nonclassical macrophages (M2) regulate tumor cells’ migration, proliferation, invasion, and angiogenesis as they contain high levels of miR-21-5p and miR-155-5p molecules [41]. These molecules have a binding site on the 3′-UTR region of the BRG1 gene (Brahma-related gene-1) and, after integrating the exosome with the tumor cell membrane, inhibit the expression of this gene. Since this gene’s product inhibits metastasis, M2 derived-exosomes increase tumor cell metastasis by inhibiting the expression of this gene [42]. Besides, miR-21-5p enhances the cellular growth and drug resistance of tumor cells by targeting PTEN (phosphatase and tensin homolog), P21 (account for cell cycle arrest), PCD (programmed cell death), and apoptotic protease activating factor 1 (APAF1) [43]. It has also been shown that miR-155-5p in M2 macrophages derived exosomes after transfer to colon cancer cells by targeting zinc-finger-type-containing 12B (ZC3H12B) leads to increased IL-6 expression, immune escape and tumor progression in colon cancer [44].

### Neutrophils-derived exosomes

Mass spectrophotometric analyses have identified 271 unique proteins within neutrophil exosomes, and their amounts vary in activated and non-activated neutrophil-derived exosomes [45]. As neutrophils are one of the first cells present at the site of inflammation, their exosomes play an important role in innate immunity. Innate immune response to infections requires the interaction of activated neutrophils with platelets which bind to circulating neutrophils via the interaction of P-selectin by glycoprotein ligand-1 (PSGL-1). This binding induces neutrophil exosome production through platelet Iba glycoprotein (GPIba). These exosomes, which contain high levels of arachidonic acid, are then transferred to intracellular structures rich in cyclooxygenase-1 (COX-1) when integrating with the platelet membrane. Exosomal arachidonic acids are converted to thromboxane A2 (TxA2) by COX-1 function. Platelet-produced TxA2 enhances the neutrophil responses by inducing endothelial cells to express intracellular adhesion molecule-1(ICAM-1), stimulating rolling, and enhancing the neutrophil migration out of vessels. Inhibition of the COX-1 function suppresses neutrophil migration and inhibits innate immunity. As a COX-1 inhibitor drug, aspirin has been widely used to reduce platelet function, which also leads to neutrophil inhibition and fever reduction [46]. Moreover, activated neutrophil-derived exosomes can interact with airway smooth muscle cells and alter their proliferative properties. Some proteins are identified in these cells’ exosomes can stimulate proliferation in their target cell, thus suggesting that these exosomes may play a role in airway structural changes in asthma [45]. Recent studies also show that neutrophil-derived exosomes by transfer miR-30d-5p induces polarization of M1 macrophages and initiate macrophage pyroptosis by activating NF-κB signaling. This study indicates that neutrophil-derived exosomes contribute to the pathogenesis of sepsis-related acute lung injury (ALI) by inducing inflammatory responses in macrophages [47].

### Mast cells-derived exosomes

Mast cell-derived exosomes carry external antigens with HSP-60 and HSC-70 as complexes and can perform diverse functions under different conditions. Mast cells can activate B and T lymphocytes by their produced exosomes under both, in vivo and in vitro conditions. External antigens present in mast cell exosomes are delivered to T cells and activates them through cross-presentation by DCs. CD91, as an endocytic receptor for this complex expressed in DCs, captures the HSP-60 or HSC-70-antigen complex. After antigen processing, these antigens are presented on cell surfaces through the MHC-II pathway. Furthermore, as mast cell exosomes contain HSP-60 and HSC-70, the expression of costimulatory molecules such as CD80, CD86, and CD40 is increased after integration with DCs. These exosomes can stimulate the maturation of DCs and converts them into better cells for antigen presentation [48]. Likewise, mast cells-derived exosomes can activate T cells through OX40L-OX40 interaction in vitro by a non-MHC-dependent pathway and stimulate their differentiation into Th2 cells [49]. These exosomes increase plasminogen activator inhibitor-1 (PAI-1) gene expression by affecting the endothelial cells due to their TNF-a precursor, angiotensin, and proteins involved in thrombin production. The product of this gene is a serine protease inhibitor and increases the risk of thrombosis and atherosclerosis [50]. Other actions performed by mast cell-derived exosomes include stimulating tumor cell proliferation in lung adenocarcinoma. These exosomes increase the expression level of cyclin D1 and accelerate tumor cell proliferation by inducing the transfer of KIT protein that binds to the SCF in the tumor cells and leads to the PI3K/AKT signaling pathway [51].

### Eosinophils-derived exosomes (EOXs)

These cells produce exosomes that have autocrine effects on the eosinophils themselves. One of the EOXs functions is to stimulate the production of reactive oxygen species (ROS) and nitric oxide (NO) by eosinophils [52]. These mediators increase inflammation by augmenting the TNF-α, CCL26, and POSTN gene expression; POSTN is a gene that can produce periostin protein and function as a ligand for some integrin receptors responsible for the eosinophilic migration. EOXs consequently prolong inflammatory conditions, leading to damage to the airway epithelium, thus playing a role in the pathology of asthma [53]. Further, EOXs carries chemotactic factors such as S100A8, S100A9, EDN, and C3a that can influence the expression of some adhesion molecules such as ICAM-1 and B1integrins family, which leads to increased cell adhesion and eosinophil migration [52]. Studies on EOXs role in the pathology of asthma have revealed that the composition of EOXs in patients with asthma and healthy individuals are similar in size and carries important enzymes such as EPO (Eosinophil peroxidase), MBP (Major basic protein), and ECP (Eosinophil cationic protein) [54]. But the critical point is that in patients with asthma, eosinophils produce more amount of exosomes compared to healthy people, which mediate tissue damage in asthma [55].

### NK cells-derived exosomes

Resting and activated NK cells constantly produce and secrete exosomes [56], and activated NK cells derived exosomes can activate non-activated NK cells. These exosomes express common NK cell markers like CD56, NKG2D, NCRs [57] and killer-related proteins such as perforin and FAS-L (membrane-bounded and soluble forms). Analyses have shown that the expression levels of perforin and FAS-L in the activated and inactivated NK exosomes are the same. Interestingly, however, the molecular weight of FAS-L is normally about 40 kDa, but in NK cells exosomes, the molecular weight of this molecule is 50 kDa [56]. The acidic microenvironment of tumors may suppress immune responses and reduce chemotherapeutic efficacy. Nevertheless, on the other hand, this acidic environment could increase the accumulation of exosomes in the tumor microenvironment, thereby enhancing the fusion of the exosome membrane with the tumor cell membrane [58]. The uptake of NK cell-derived exosomes by tumor cells induces apoptosis through perforin and Fas-L in them [59]. Likewise, NK cell exosomes play a cytotoxic role against the activated immune cells, thereby maintaining the homeostasis of immune responses [60]. The Yutaka Enomoto et al. study results show that stimulation of NK cells by a combination of IL-15 and IL-21 leads to an increase in their cytotoxic activity. However, further studies have shown that this increased cytotoxic capacity is not related to cytotoxic granules and is mediated by their EVs, including exosomes. Finally, mass spectrometric analyzes in different experimental groups showed that the level of CD226 (DNAM-1) on the surface of activated NK cells (by IL-15 + IL-21) derived exosomes was significantly increased. These exosomes are captured by tumor cells through macropinocytosis, and blocking CD226 (using monoclonal antibodies) reduces the cytolytic activity of activated NK cells exosomes and EVs [61].

### B lymphocytes derived-exosomes

B lymphocytes activated through B cell receptor (BCR) or TLR secrete more exosomes than the inactivated B cells [62]. These exosomes express MHC-I and II and CD40 CD54, CD63, CD80, CD81, and CD86. In addition, B cell-derived exosomes express CD19, which is a specific marker of B cells [63]. Also, they carry the MHCII-peptide complex and stimulate T lymphocytes activation through binding to them. Similarly, these exosomes can bind to follicular dendritic cells (FDCs) as they contain α4β1 integrins that interact with VCAM-1 on the surface of FDCs. These cells do not produce MHC-II molecules, but they are observed in inactive and secondary forms on FDC surface. After binding of B lymphocyte-derived exosomes to FDCs, MHC-II molecules are expressed by dressing phenomenon on their surfaces. This molecule on the FDC surface is necessary to clonal select in the secondary follicle light zone to induce the affinity maturation phenomenon in B lymphocytes [64]. These exosomes also express β1 and β2 integrins. By the expression of β1 integrins, they can bind to TNF-a-activated fibroblasts and collagen type I and fibronectin in the extracellular matrix (ECM) [65]. Some studies have shown that B lymphocyte exosomes can directly stimulate T lymphocytes activation. Still, B cell-derived exosome-mediated activation of T cells has led to the production of cells that have little proliferation and cytokine production compared to the usual mode of T lymphocyte activation [66].

In contrast, other studies have shown that the activatory potential of B cell-derived exosomes is due to their interaction with APCs and their stimulation [27]. Direct activation of T lymphocytes by B lymphocyte exosomes depends on several factors, namely the affinity of T cell receptor (TCR) to the MHC-II-peptide complex on exosomes, the concentration of exosomes, the expression levels of costimulatory molecules on the surface of exosomes, and also the cell type and phenotype of exosome origin and target cells [63]. Environmental changes can affect the B lymphocytes derived exosomes’ internal cargos. For example, in response to heat stress, B cells produce exosomes with a high amount of heat shock proteins (HSPs) such as HSP-27, HSC-70, HSP-70, and HSP-90 increases [67].

### T lymphocytes-derived exosomes

Naïve T lymphocytes produce exosomes that modulate the activity of immune cells and express the specific TCR and various adhesion molecules [68]. Besides, T cell exosomes express other markers such as CD3, CD2, CD4, CD8, CD11c, CD25, CD69, LFA-1 (lymphocyte function-associated antigen1), CXCR4, FASL, and GITR (glucocorticoid-induced TNF receptors family-related gene) [69]. Exosomes produced from activated T-cell with IL-2 increase the proliferation of resting autologous cells [70]; they carry a large amount of microRNAs and affect the functions of target cells upon integration with them. Immunological synapses enhance T cell exosome transferring efficacy between T-cell and APCs. During this process, T cell exosomes convert APCs into better cells for antigen-presenting by transferring miRNAs [71]. Exosomes produced from activated CD8^+^ T cells can activate ERK and NF-κB signaling pathways in melanoma cells, increasing the metastasis of cancerous cells in vitro by increasing matrix metalloproteinase-9 (MMP9) expression [72].

Regulatory T lymphocytes (Treg) produce and secrete large amounts of exosomes which express markers such as CD25, CTLA-4, and CD73 and suppress the immune system through various mechanisms. CD73, on the surface of these exosomes, plays an important role in suppressing immune system responses by inducing adenosine production (role in the anti-inflammatory response) [73]. Treg exosomes prevent the formation of Th1 and IFN-γ production by transferring let-7b, let-7d, and microRNA-155 to the target cells. Likewise, many of the inhibitory functions of these exosomes are due to the presence of IL-10 and TGF-β cytokines [74]; Treg derived exosomes can also regulate the function of DCs by transmitting miRNAs. These exosomes increase the production of IL-10 by reducing the production of IL-6 and TNF-a by transferring miR-150-5p and miR-142-3p to DCs [75].

Microarray analyses have identified 257 different types of lncRNAs in exhausted and non-exhausted TCD8^+^ cell exosomes. Functional analyses show that these lncRNAs regulate various TCD8^+^ processes, such as metabolism and gene expression. Since exhausting and normal T cell exosomes have different lncRNAs, their function is different. Exhausted T cell exosomes are captured by the normal TCD8^+^ cells and alter their function [76]. During this transfer, the rate of proliferation and production of cytokines in non-exhausted TCD8^+^ cells decreases, and the levels of inhibitory markers such as PD-1 and TIM-3 increase at their surfaces [76, 77]. Because lncRNAs are the main factor in this action, exosomes must integrate with the target cell membrane [78].

Due to the associated problems with CAR-T cell therapy, namely CAR-T cell cytokine release syndrome (CRS), which can lead to hypotension, nausea, tachycardia, and headache [79], some researchers have suggested the application of their exosomes as therapeutic agents. Because normal T-cell exosomes express TCR, it is suggested that CAR-T cell exosomes can also express the chimeric receptor and mimic CAR-T cell receptor characteristics; they have advantages over the use of CAR-T cells. First, they can transfer granzyme to the target cell along with lysosomal enzymes without the need for perforin by fusion with the target cell or via endocytosis [80]. Second, exosomes being very small in size, can cross the blood-brain barrier and tumor-forming blood barrier and perform their functions on the target cell [81]. Third, as antitumor drugs can be loaded into these exosomes, the effectiveness of the combat against the tumor is significantly increased as these exosomes can deliver anticancer drugs specifically to tumor cells [82]. In addition to the above, exosomes tend to bind to the ECM of solid tumors, which increases their therapeutic efficacy [72].

**Fig. 2:**
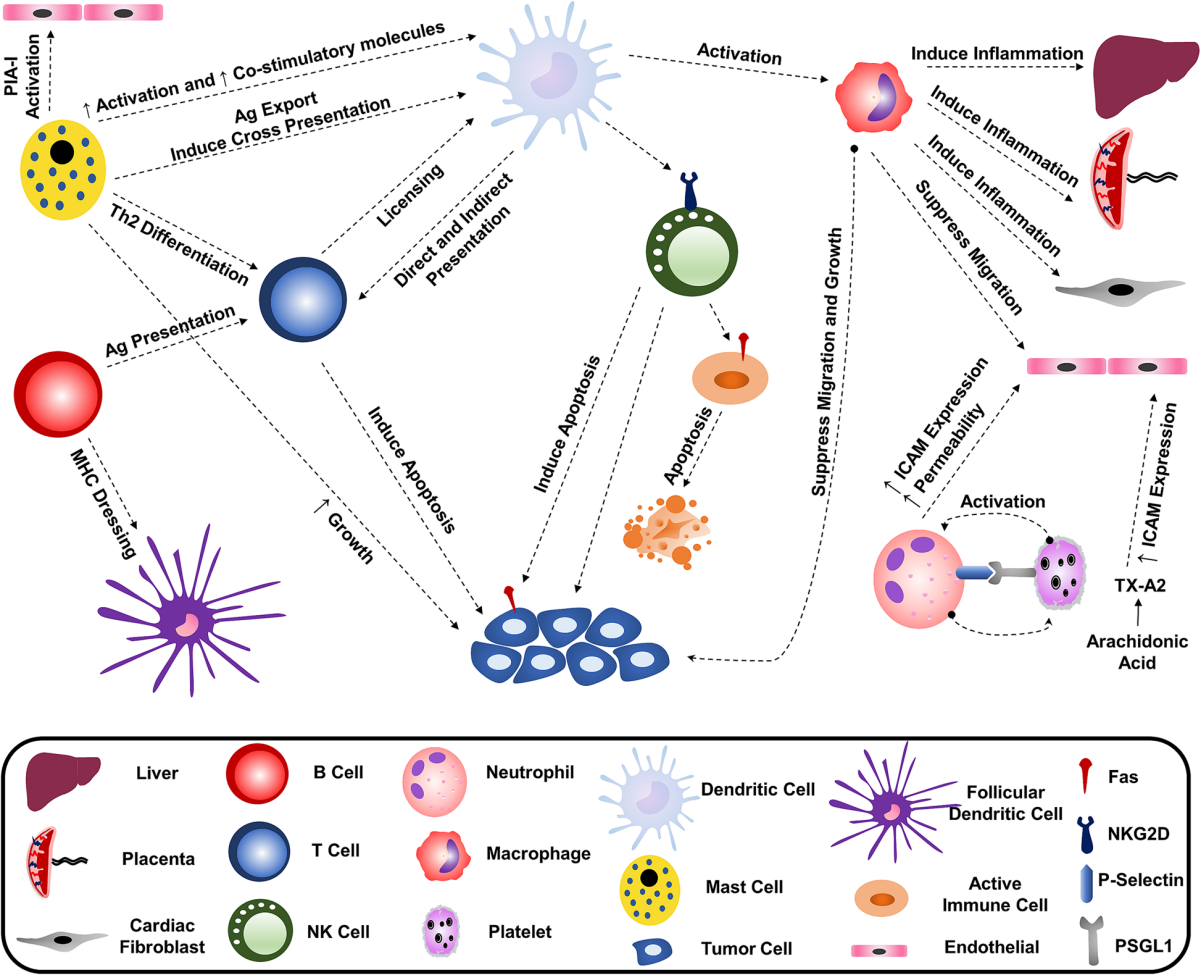
Interrelationship between immune cells by producing exosomes. Each immune cell secret exosomes and release them into the microenvironment. These exosomes can affect other immune cells’ functions by suppressing or activating them and so affects immune responses

According to the above, exosomes derived from each immune cell can affect the function of other immune and non-immune cells. Figure 2 summarizes the intercellular communication of immune system cells by exosomes and their effects (Fig. 2).

## Role of immune-cell derived exosomes in diseases

Immune system cells play a crucial role in the pathogenesis of various diseases by producing exosomes. Exosomes from different cell sources transmit different cargos to the target cell and can change their behavior and functions. Since many of these cells trigger inflammatory responses to remove pathogens, their exosomes can play a role in inflammatory diseases such as tissue destruction, autoimmunity, and allergies. Further, some immune cells may help modulate responses and homeostasis by suppressing immune responses. These regulatory cells produced exosomes can contribute to the development of cancer and infectious diseases. Table 1 summarizes the role of IEXs in the pathogenesis of various diseases (Table 1).

**Table 1:** The role of IEXs in the pathogenesis of various diseases

Exosome source	mediator	Target cell	Mediator target	Type of disease	Result
M2 macrophages	miR-221-3p	Tumor cells	Cyclin-dependent kinase inhibitor 1B	Tumor	Resolve the cell cycle arrest in the G1/S and help tumor cells proliferation
M2 macrophages	miR-29a-3p and miR-21-5p	TCD4^+^ cells	STAT3	Tumor	1. These exosomes disrupt the Treg/Th17 balance 2. ↓ Inflammatory cytokines production such as IL4, IL6, and TNF-a 3. ↑ Anti-inflammatory cytokines production like IL10
M2 macrophages	miR-21-5p and miR-155-5p	Tumor cells	Brahma-related gene 1 (BRG1)	Tumor	↑ Colon cancer cells’ migration and metastasis
Monocyte-derived macrophages	miR-223	Tumor cells	Myocyte-enhancing factor-2c (Mef-2c)	Tumor	1. ↑ β-catenin nuclear accumulation 2. ↑ Breast cancer cells’ migration and metastasis
M2 macrophages	Apolipoprotein E (ApoE)	Tumor cells	PI3K/Akt/mTOR signaling pathway	Tumor	↑ Gastric cancer cells’ migration and metastasis
Myeloid-derived suppressor cells (MDSCs)	Fas-L (CD95L)	TCD8^+^ cells	Fas (CD95)	Tumor	Induce activation-induced apoptosis (AICD) in TCD8^+^ cells
M2 macrophages, Dendritic cells (DCs), and T lymphocytes	miR-21	Tumor cells	APAF1, PTEN, PDCD4, and P21	Tumor	1. ↓ Caspases 9 and 3 activations and apoptosis initiation 2. ↑ Drug resistance in cancer cells
M2 macrophages	non-coding RNA that stabilizes HIF-1α (HISLA)	Tumor cells	Hypoxia-inducible factor 1-alpha (HIF-1α)	Tumor	1. Inhibits hydroxylation and degradation of HIF-1α 2. ↑ Aerobic glycolysis and enhanced resistance to apoptosis in cancer cells
DCs	MHC-I and II	Alloreactive T cells	N/A	Tissue transplant rejection	Stimulate the anti-alloantigen responses
DCs and macrophages	leukotriene A4 (LTA4), LTB4 and LTC4	Immune cells, especially neutrophils	N/A	Asthma and allergic diseases	↑ Inflammation by attracting other immune cells
Thymic stromal lymphopoietin (TSLP)-activated DCs	OX40-L	TCD4^+^ cells	OX40	Asthma and allergic diseases	1. ↑ TCD4+ cells differentiation to Th2 2. ↑ IgE production
Mast cell	CD40L	B lymphocytes, T lymphocytes, bronchial smooth muscle cells (BSMCs)	CD40	Asthma and allergic diseases	1. Stimulate IgE production 2. Stimulate the production of pro-inflammatory cytokines
HTLV-1 infected T lymphocytes	Tax proteins and viral mRNAs such as Tax, HBZ, and Env	Different types of host cells	Activate the NF-κB, and Akt pathways, stimulating ROS production	Infectious diseases	1. ↑ Infected cell survival 2. Contribute to the spread and pathogenesis of HTLV-1-induced disease 3. Disrupts DNA repair mechanisms
T lymphocytes	miR-155, miR-142-5p, and miR-142-3p	Pancreatic beta cells	Regulate the NF-κB related signaling pathway	Type 1 diabetes	↑ Secretion of CCL2, CCL7, and CXCL10 and apoptosis in pancreatic beta cells
Adipose tissue-derived macrophages	N/A	Macrophages	Reduce the insulin-dependent phosphorylation of Akt by increasing NF-κB activation	Type 2 diabetes	1. ↓ The transfer of GLUT4 to the cell surface 2. Blocks glucose uptake 3. Stimulating M1 macrophage differentiation
M1 macrophages	N/A	TCD4^+^ cells and CD8+ T cells	Regulate the T-bet transcription factor activity	Guillain-Barré syndrome	1. ↑ Th1 cells responses 2. ↑ The ratio of IFN-γ and IL-17 producing CD4^+^ T cells
T lymphocytes	miR-326	Inhibit Ets-1 and CD47 expression	TCD4+ cells, monocytes, brain resident cells	Multiple Sclerosis	1. ↑ Th17 differentiation in vitro and in vivo 2. ↑ ICAM-1 and Mac-1 (macrophage-1 antigen) expression in monocytes 3. ↑ Phagocytic activity of macrophages

*ICAM-1* Intercellular adhesion molecule 1, *APAF1* Apoptotic peptidase activating factor 1, *PTEN* Phosphatase and tensin homolog, *NF-κB* Nuclear factor-kappa B, *NK cell* Natural killer cells, *TNF-α* Tumor necrosis factor-alpha, *IL* Interleukin, *MCP-1* Monocyte Chemoattractant Protein-1, *OVA* Ovalbumin, *MHC* Major histocompatibility complex, *TLR* Toll-like receptor, *AchR* Acetylcholine receptor, *TGF-β1* Transforming growth factor-beta 1, *N/A* Not applicable

### Role of IEX in cancer

In the tumor microenvironment, diverse cells such as immune cells actively interact with their surroundings through EVs and facilitate tumor malignancy by regulating malignant cascades such as immune modulation, angiogenesis, drug resistance, cell proliferation and metastasis [21]. Immune cells located in these environments are altered by exosomes derived from tumor cells and contribute to tumor progression [21, 83].

#### Role of IEX in tumor enlargement by increasing tumor cell proliferation

Microarray analyses have shown that M2 macrophages contain large amounts of miR-21-5p, miR-24-3p, and miR-221-3p. Because miR-221-3p directly suppresses the cyclin-dependent kinase inhibitor 1B (CDKN1B), these exosomes can resolve the cell cycle arrest in the G1/S and help tumor cells proliferate. Furthermore, in vivo analyses indicate that M2 macrophage-derived exosomes play a role in epithelial ovarian cancer (EOC) [84].

#### Role of IEX in preventing inflammatory responses in immune cells

Treg/Th17 cell imbalance occurs in many diseases such as human systemic lupus, diabetes, and some tumors. As mentioned, M2 macrophage exosomes contain a variety of miRs. A study by Zhou et al. shows that M2 macrophage exosomes transfer miR-29a-3p and miR-21-5p (with a synergistic mechanism) to TCD4^+^ cells. These exosomes disrupt the Treg/Th17 balance by stimulating the differentiation of Naive cells to Treg by inhibiting the STAT3 transcription factor. During this procedure, the production of inflammatory cytokines such as IL4, IL6, and TNF-a decreases, and anti-inflammatory cytokines like IL10 increase. Thus, these exosomes suppress T cell anti-tumor responses by stimulating the differentiation of TCD4^+^ cells into Treg and contribute to tumor progression [85].

Myeloid-derived suppressor cells (MDSCs) in the tumor microenvironment are more immune suppressive than MDSCs in peripheral lymphatic organs [86]. In vivo and in vitro analyses show that exosomes produced from MDSCs can suppress immune system cells such as macrophages and T cells in the tumour environment. A study by Rashid et al. found that the use of MDSC exosomes increases activating markers (CD69, CD44) and also exhausting markers (PD-1/CD279) by repetitive stimulation of TCD8^+^ cells, which leads to induction of a state of non-responsivity (exhausting). Repetitive activation of TCD8^+^ cells leads to increased ROS production in these cells. Furthermore, since MDSC exosomes express high Fas-L levels at their surface, they can induce activation-induced apoptosis (AICD) in TCD8^+^ cells. Thus, in general, MDSCs-derived exosomes inhibit TCD8^+^ cell function by inducing exhausting conditions, ROS production, and apoptosis induction through Fas/FasL (CD95/CD95L) interaction and contributing to tumor tumours progression [87].

#### Role of IEX in the migration/invasion of cancer cells

The M2 macrophage-derived exosomes carry miR-21-5p and miR-155-5p, enhance colon cancer cells’ migration and metastasis, and contribute to tumor progression. These miRs inhibit the expression of Brahma-related gene 1 (BRG1) by binding to this gene. Since BRG1 plays a vital role in inhibiting these cells’ metastasis, its inhibition leads to increased metastasis and invasion of colon tumor cells [42]. Yang et al. illustrated that macrophages derived from monocytes treated with IL-4 produce exosomes containing larger amounts of miR-223 and inhibit the expression of myocyte-enhancing factor- 2c (Mef-2c) by binding to 2 target sites in 3′-UTR after transfer to a breast cancer cell. Because Mef-2c depletion is associated with β-catenin nuclear accumulation, the rate of cell migration and metastasis in breast cancer cells increases [88]. Mass spectrometric analyses of M2 macrophages-derived exosomes indicate apolipoprotein E (ApoE) on their surface and increase metastasis in gastric cancer cells after their fusion and transfer of ApoE to them. ApoE is an important regulator of tumor progression through increased cell adhesion, proliferation, and migration with cytoskeletal rearrangement through the PI3K/Akt/mTOR signaling pathway [89].

The TCD8^+^ cells in the tumor microenvironment are transformed into a non-responsive state through factors secreted by the tumor cells. Cai et al. demonstrated that these cells produce FasL^+^ and CD8^+^ exosomes in the tumor microenvironment and enhance the metastasis in the B16 melanoma cell line. These exosomes activate the NF-κB, ERK, and c-FLIPL signaling pathways through the Fas/FasL-dependent trail, leading to MMP2, MMP9, and COX2 increased expression in B16 cancer cells, which play a critical role in cancer cell metastasis [72].

#### Role of IEX in drug resistance and reduction of tumor cell apoptosis

M2 macrophages, DCs, and T cells derived exosomes carry large amounts of miR-21, which can induce drug resistance in cancer cells by binding to APAF1. The binding of APAF1 to cytochrome c leads to caspases 9 and 3 activations and apoptosis initiation. Thus, the inactivation of APAF1 function can lead to drug resistance in cancer cells. However, APAF1 cannot be entirely responsible for the effect of miR-21 on increasing paclitaxel resistance in ovarian cancer [43]. Other studies have shown that miR-21 can increase the survival and drug resistance to tamoxifen, faslodex, and topotecan in breast cancer therapy by targeting other genes such as PTEN, PDCD4, and P21 [90].

A 2019 study by Chen et al. showed that M2 macrophage derived-exosomes contain a lncRNA named long non-coding RNA that stabilizes HIF-1α (HISLA). These exosomes inhibit the interaction between the prolyl hydroxylase 2 (PHD2) and hypoxia-inducible factor 1-alpha (HIF-1α) and prevent the hydroxylation and degradation of HIF-1α after fusion with breast cancer cells via HISLA transfer [91]; HIF-1α is an oxygen-sensing transcription factor that determines the type of glucose consumption by oxidation or glycolysis [92]. Therefore, increasing the level of HIF-1α leads to increased aerobic glycolysis and enhanced resistance to apoptosis in cancer cells [93].

### Role of IEXs in transplant rejection

EVs, including exosomes, play an important role in transplant rejection by delivering antigens to immune cells. Exosomes produced by APCs are abundant in MHC and T cell-associated adhesion molecules on their surface. Allograft derived exosomes (Allo-Exo) released by donor leukocytes “throw out” from the connective tissue and pass through the pores of the recipient’s lymphatic capillaries into the transplanted drainage lymph tissue [94].

Many studies show that donor-derived DCs levels in transplanted lymph nodes are undetectable in acute allograft rejection; therefore, it is likely that other mechanisms are involved in this type of transplant rejection. Recent studies have shown that donor DEXs mimic the characteristics of DCs (having MHC-I and MHC-II and helper molecules) and migrate to the draining lymph node, and present the MHC to alloreactive T cells to stimulate the anti-alloantigen responses [95].

Exosomes have a limited ability to activate T cells due to the low number of MHC-peptide complexes and costimulatory molecules as APCs capture them in phagocytosis or other ways [96]. These cross-dressed APCs express exosome-derived MHC-peptide complexes on their surface and activate alloreactive T cells with higher efficiency [97]. Recent studies show that the innate immune system cells are also involved in transplant rejection, wherein monocyte-derived DCs are produced continuously to participate in the transplant rejection process [98] in mice with defective T cells, B cells, and innate lymphocytes. Since Allo-Exo’s main role in transplant rejection has not been precisely elucidated, several actions can be attributed to them, such as increasing macrophage phagocytic activity, providing antigen for presentation, and stimulating the monocytes’ maturation to inflammatory DCs [99, 100].

### IEXs role in the pathogenesis of asthma and allergic diseases

Asthma is a chronic inflammatory disease of the respiratory tract with a complex pathophysiology and is classified as a type 1 hypersensitivity disease [101]. Exosomes play an important role in the pathogenesis of asthma and intercellular signaling by transporting various cargoes to target cells. They can increase inflammation by regulating the immune cells’ function (increasing recruitment, activation, or differentiation) [102].

Previous studies have shown that DCs and macrophages derived exosomes contains lipid mediators derived from arachidonic acid such as leukotriene A4 (LTA4) [54] and participate in the pathogenesis of some allergic diseases and chronic inflammation. Besides, DEXs contain enzymes that can convert LTA4 to other leukotrienes (LTs), such as LTB4 and LTC4. Since LTB4 is also a chemotactic factor, it increases inflammation by attracting other immune cells to the inflammation site [5]. Thymic stromal lymphopoietin (TSLP)-activated DCs also induce T cell responses and their differentiation to Th2 through the interaction between OX40-OX40-L with T cells, which can help in IgE production [103]. Lymphocytes are key players in the inflammatory response in allergies and asthma. B cell-derived exosomes present allergen-derived peptides such as birch peptide (Bet v1) to T cells and induce their differentiation to the Th2 phenotype [63]. Mast cell exosomes stimulate IgE production in B cells through the CD40-CD40L interaction in the absence of T cells. Besides, it has been shown that these exosomes can stimulate the production of pro-inflammatory cytokines by T lymphocytes and bronchial smooth muscle cells (BSMCs) and contribute to the progression of asthma [48].

### Role of IEXs in the progression of infectious diseases

Exosomes produced by infectious agents can lead to the spread of infection and prevent immune system responses [104]. Also, infected cells with intracellular pathogens can secrete exosomes that play an important role in the infection’s fate. They can directly carry damaging pathogen related molecules and contribute to infectious disease pathology, thus leading to the spread of infectious diseases [105].

Human T-lymphotropic virus type 1 (HTLV-1) infected T cells produce exosomes containing various viral proteins that contribute to the pathogenesis of HTLV-1-induced disease. These exosomes contain Tax proteins and transcripts of viral mRNAs such as Tax, HBZ, and Env [106], which are translated into proteins by the host cell and exert their functions; Tax is an important protein for regulating the dynamics of the viral life cycle and regulating various genes and can activate the NF-κB, and Akt pathways which is two main cell survival pathways in target cells.

**Fig. 3:**
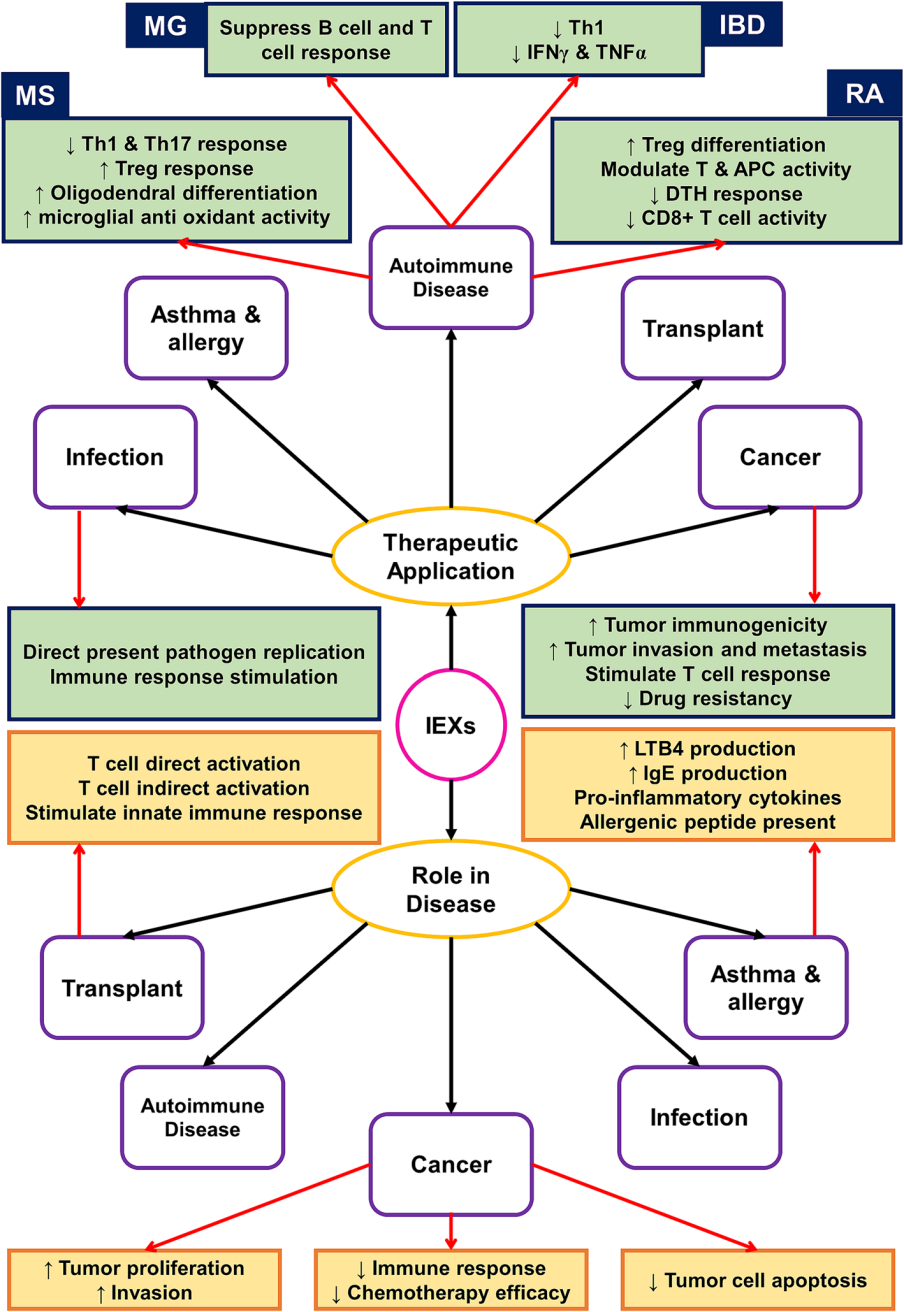
IEX has a role in the pathogenesis of the disease. Also, these exosomes play a role in various disease treatments. As shown in the figure, IEX therapeutic application is an exciting subject used for autoimmune disease, cancer, asthma, allergy, transplantation, and infection treatment

Furthermore, Tax can play a role in the disease’s pathogenesis by damaging cellular DNA in two ways. This molecule disrupts DNA repair mechanisms such as base exchange repair (BER), nucleotide exchange repair (NER), and mismatch repair (MMR). Also, Tax directly damages cellular DNA by stimulating ROS production in host cells. Thus, exosomes produced from HTLV-1 infected T cells can lead to genetic damage in other Naive T cells and prevent their function [107]. HIV-1 infected T cells also produce exosomes containing the p24 and Nef proteins and a double-stranded non-coding RNA named TAR-RNA. These exosomes can transfer viral related proteins to adjacent cells and contribute to the pathogenesis of the disease; Nef is one of the HIV-1 associated proteins and helps to virus replication and progression of the disease [108]. Monocytes are among the most important sources of HIV-1 infection by this virus. Analyses results show that Nef-expressing monocytes produce AATK, SLC27A1, and CDKAL mRNAs containing exosomes. Transfer of This mRNA to surrounding TCD4^+^ cells induces apoptosis, disrupts fatty acid metabolism, and thus participates in the pathogenesis of HIV-1 [109]. Another study showed during infection with human herpesvirus-6 (HHV-6), infected cells such as TCD4^+^ [110] produce exosomes that have a number of complete and mature viruses and are transmitted to other cells, thus, leading to the spread of infection [111]. Figure 3 summarizes the role of IEXs as double-edged swords in disease progression as well as their therapeutic applications (Fig. 3).

### Role of IEXs in the pathogenesis of autoimmune disease

#### Type 1 diabetes (T1D)

Guay et al. showed that human and rodent T cells derived exosomes carry the miR-155, miR-142-5p, and miR-142-3p, which can stimulate the secretion of different chemokines including CCL2, CCL7, and CXCL10 and regulate the NF-κB related signaling pathway and apoptosis in pancreatic beta cells in T1D. These chemokines may promote immune cells’ recruitment and increase the selective death of insulin-secreting cells. Inactivation of these miRs in NOD mice increases insulin levels, decreases insulitis scores and inflammation, and also prevents the progression of diabetes [112].

#### Type 2 diabetes (T2D)

Zhang et al. found that cytokines secreted by adipose tissue macrophages (ATMs) significantly altered the adipocyte function, inducing inflammatory responses and stimulating M1 macrophage differentiation. Exosomes derived from these macrophages in the presence of insulin in adipose tissue reduce the insulin-dependent phosphorylation of Akt by increasing NF-κB activation. This action blocks glucose uptake by reducing the transfer of GLUT4 to the cell surface. Thus, these exosomes increase insulin resistance due to obesity in adipose tissue, which is a significant component in T2D pathogenesis [113].

#### Guillain-Barré syndrome

Du et al. found that M1 macrophages-derived exosomes intensified the autoimmune neuritis (EAN), the animal model of Guillain-Barré syndrome, by increasing Th1 responses. Guillain-Barré syndrome is the most common acute paralysis neuropathy, which results from an immune system attacking the peripheral nervous system and demyelination of neurons [114]. M1 macrophage exosomes in the in vivo studies directly increase the ratio of IFN-γ and IL-17 producing CD4^+^ T cells in the lymph nodes and spleen, which promotes EAN progression. Evidence also suggests that these exosomes induce the expression of pro-inflammatory cytokines such as IL-6 and IL-12 in DCs and macrophages, which in turn amplifies the Th1 and Th17 responses via indirect pathways. These exosomes induce in vitro proliferation, differentiation, and effective function in CD4^+^ and CD8^+^ T cells that produce IFN-γ. Experimental results suggest that M1 macrophage-derived exosomes may affect T cells by regulating the T-bet transcription factor [115].

#### Multiple sclerosis (MS)

Studies show that exosomes secreted by T cells contain large amounts of miR-326, which, by targeting and suppressing the Ets-1 gene product as a negative regulator of Th17 differentiation, increases the Th17 differentiation in vitro and in vivo and thus exacerbates MS [116]. Studies show that microglia cells secrete exosomes that contain IL1-β, MHC-II, MMPs, and caspase-1 into the cerebrospinal fluid, which can cross the blood-brain barrier (BBB). These exosomes are distributed throughout the body and contribute to antigens’ rapid release and their delivery to immune cells. The enzymes in these exosomes damage the BBB and cause neuroinflammation by immune system cells migrating increase, resulting in MS. Exosomes derived from active T cells increase ICAM-1 and Mac-1 (macrophage-1 antigen) expression in monocytes. The expression of these two molecules increases the migration of active monocytes and intensifies MS [117]. It has also been shown that some of the miRs involved in the pathogenesis of MS in T cell-derived exosomes, such as miR-326, are significantly higher than in healthy individuals [116]. miR-326 helps to promote MS and myelin degradation by inhibiting CD47 expression in brain resident cells that inhibit the phagocytic activity of macrophages [118].

## Immune cell-derived exosomes (IEX) - therapeutic applications

The ability of IEX to enhance or suppress immune responses has led to their therapeutic usage. Because in certain diseases, the function of some immune cells and the production of exosomes from them increases, they can be used as diagnostic markers [6]. Also, as mentioned earlier, the exosomes from different cells of the immune system cells have dissimilar contents that lead to different responses by affecting the target cells. These exosomes can be isolated from the patient’s immune cells and be injected as an autologous graft. Table 2 summarizes some new studies pertaining to IEXs and their deployment in treating various diseases (Table 2).

**Table 2:** New studies in the field of IEXs therapeutic application

Study name	source	year	ref
Anti-CTLA-4 antibody-functionalized dendritic cell-derived exosomes targeting tumor-draining lymph nodes for effective induction of antitumor T-cell responses	DC	2020	[119]
Exosome-Derived circITGB1 Regulates Dendritic Cell Maturation and Cardiac Inflammation via miR-342-3p/NFAM1	DC	2020	[120]
Dendritic cell derived exosomes loaded with immunoregulatory cargo reprogram local immune responses and inhibit degenerative bone disease in vivo	DC	2020	[121]
M1-like macrophage-derived exosomes suppress angiogenesis and exacerbate cardiac dysfunction in a myocardial infarction microenvironment	M1 macrophage	2020	[122]
M2 macrophage-derived exosomes promote the c-KIT phenotype of vascular smooth muscle cells during vascular tissue repair after intravascular stent implantation	M2 macrophage	2020	[123]
Neutrophil-derived exosome from systemic sclerosis inhibits the proliferation and migration of endothelial cells	Neutrophil	2020	[124]
miR103a-3p in exosomes derived from human mast cells (MCs) following aggregation of FcεRI enhances IL-5 production from IL-33-stimulated type2 innate lymphoid cells (ILC2) via silencing protein arginine methyltransferase (PRMT)	Mast cells	2020	[125]
Eosinophil-Derived Exosomes Contribute to Asthma Remodeling by Activating Structural Lung Cells	Eosinophil	2018	[126]
NK cell-derived exosomes carry miR-207 and alleviate depression-like symptoms in mice	NK cell	2020	[127]
Treatment of cancer and infectious diseases with natural killer (nk) cell-derived exosomes	NK cell	2020	[128]
Role of Chronic Lymphocytic Leukemia (CLL)-Derived Exosomes in Tumor Progression and Survival	B cell	2020	[129]
Exosomes derived from regulatory T cells ameliorate acute myocardial infarction by promoting macrophage M2 polarization	Treg	2020	[130]
CAR exosomes derived from effector CAR-T cells have potent antitumour effects and low toxicity	CAR-T	2019	[131]

*DC* Dendritic cell, *CAR-T cell* Chimeric antigen receptor expressing T cell, *Treg* Regulatory T cell

### IEX therapeutic application in cancer

Tumor tissue and cells have evolved to suppress immune responses. The tumor microenvironment contains molecules and factors that suppress the immune system and prevent the development of DCs and T lymphocytes. Therefore, establishing specific, strong, and long-lasting immune responses against cancer cells and reducing immune suppressants are critical for cancer effective treatment. The results of a study by Pete et al. show that the use of DEXs has several advantages over DCs applications in cancer immunotherapy [32, 132]. At present, exosomes are preferred to cell therapy for Five reasons. (1) These vesicles can be stored for a long time without losing immune activity; (2) Exosomes, due to their membranes’ similarity to body cells, integrate with the target cell more efficiently than soluble factors produced by cells [133]; (3) They are smaller than cells and can easily pass through different capillaries [134]; (4) Ability to easily manipulate exosomes and produce engineered exosomes [135]; (5) Easier injection compared to cell therapy (possibility of nasal administration) [135].

#### Transformation of cancer cells into cells with higher immunogenicity

Because DEX carries many DC-related immune-stimulating molecules, tumor cells’ uptake can turn tumor cells into better immune targets. A study by Romagnoli et al. showed that in vitro treatment of SK-BR-3, U87, and K562 cell lines by mature DCs exosomes is associated with increased expression levels of MHC-I, MHC-II, CD86, CD11c, CD54, and CD18 in them. This makes the tumor cells more suitable to activate the immune system. Interestingly, when DEXs are exposed to anti-CD9 antibodies before treatment, they fuse to a lesser extent with tumor cells, indicating this molecule’s role in integrating the exosome with the tumor cell [136]. Human breast adenocarcinoma cells (SK-BR-3) treated with DEX could be used to stimulate CD3^+^ T cells. It has been shown (by ELISPOT analysis) that the number of IFN-γ-producing T cells in the DEX-treated tumor cells group is much more than in the untreated control group. These data suggest that treating tumor cells with DEXs increases their ability to activate T cells for a more efficient response [137].

#### Stimulation of tumor antigen-specific T cells responses

DEXs carry the molecules needed to activate tumor-specific immune cell response and perform as an anti-tumor vaccine alone [138]. For this purpose, DCs are first isolated from patients, and after activation with tumor-specific antigen and proliferation, these cells are injected via various routes. It has been shown that DEXs can be used as acellular vaccines that express melanoma-associated antigens (MAGE) on MHC-I and MHC-II. In a study, IFN-γ-stimulated monocytes derived exosomes increased the activity of NK cells and were associated with tumor regression and recovery in patients with lung cancer [139].

In a study by Lu et al., it has been shown that alpha phytoprotein (AFP) activated DCs derived exosomes can stimulate antigen-specific immune responses and increase tumor regression in mice with hepatocellular carcinoma (HCC) [140]. Also, the measurement of splenic IFN-γ producing TCD3^+^ cells by fluorescence-activated cell sorting (FACS) reveals a significant increase in the number of these cells in the DEX-AFP-treated group compared to the control group. This study illustrated that T cells’ production of inflammatory cytokines such as IFN-γ and IL-2 has increased, and the level of inhibitory cytokines such as TGF-β1 and interleukin-10 increased as well [140, 141]. Stimulation of DCs by TLRs ligands such as poly I:C (TLR-3 ligand), LPS (TLR-4 ligand), and CpG DNA (TLR-9 ligand) lead to the production of DEXs that activate the immune system and significantly increase their ability for anti-tumor therapy. The use of melanoma B16F10 cell lysates combined with poly (I: C) to stimulate DCs results in DEX production that can stimulate CD8^+^ T cells; these exosomes also increase recruitment cytotoxic CD8^+^ T, NKT, and NK cells to the tumor site. As a result, tumor growth is significantly reduced, and the patient’s survival rate increases [142].

Martina Damo et al. reveal that exosomes isolated from matured DCs with albumin purified antigen and poly (I:C) antigens (DEX (OVA + pIC)) supernatant stimulate more robust Th1 responses by secretion of IFN-γ and TNF-α as proinflammatory cytokines, compared to exosomes isolated from DCs matured by LPS and OVA (DEX (OVA + LPS)) as well as DCs matured by CpG and OVA (DEX (OVA + CpG)). These cellular immune responses stimulate the tumor regression in the absence of OVA-specific antibodies, indicating that DEXs do not play a role in producing tumor-specific antibodies [142]. Another study by Chen et al. confirmed the results of a previous investigation wherein HPV E7_49-57_ antigens in combination with poly (I:C) had been used to stimulate DCs maturation to produce DEX (Dex (E7 + pIC)) for the treatment of cervical tumors. The use of this Dex (E7 + pIC) based vaccine induces a specific CTL response against E7_49-57_ [143].

#### Stimulation of apoptosis in tumor cells

Previous studies have shown that hematopoietic and tumor cells may secrete HSPs into the bloodstream through granulomas, exosomes, or exocytosis [144]. These extracellular HSPs stimulate innate immune responses through TLRs [145]. Treatment of DCs with CO_2_ hyperthermia (HT-CO_2_) results in exosome production in which HSP70 levels are elevated. Evaluation of these exosomes’ effect by analyzing the activity of caspase-3 and measuring CCK-8 indicates an increase in apoptosis and a decrease in proliferation in the AGS cell as a gastric cancer cell line [146].

miR-let-7a-5p is present in large amounts in the M1 macrophages exosomes, and this miR targets four genes, including BCL2-like protein 1 (BCL2-L1), insulin-like growth factor 1 receptor (IGF1R), mitogen-activated protein kinase 8 (MAPK8), and Fas [147]. These genes are functionally divided into two groups involved in autophagy, including BCL2-L1, IGF1R, and MAPK8, and the group of genes involved in apoptosis, encompassing BCL2-L1, Fas, and MAPK8. Duan et al. showed that let-7a-5p decreased BCL2-L1 gene expression by stimulating the PI3Kγ signaling pathway and increasing autophagy and apoptosis in lung cancer cells [148].

NK cell exosomes express the DNAX-1 accessory molecule (DNAM1) on their surface, enabling exosomes to integrate with the tumor cell membrane by binding to PVR and Nectin-2. Integration of NK exosomes containing perforin and granzyme leads to the onset of apoptosis in the NALM-18 cell line (associated with acute lymphoblastic leukaemia in childhood) due to caspase-8 activation [149]. Exosomes derived from NK cells also contain granulysin that disrupts the tumor cell membrane integrity [150]. These exosomes induce apoptosis in tumor cells by damaging the mitochondria and activating caspase 9 and 12 [151]. In a study by Jong and colleagues, it has been shown that NK cell exosomes induce several caspase pathways by using different cytotoxic molecules in SupB15 cells associated with acute lymphoblastic leukaemia (ALL) and neuroblastoma cancer cell lines [152].

#### Inhibition of metastasis, migration, and proliferation in tumor cells

Given that approximately 70% of ovarian cancer cells express high levels of epidermal growth factor receptor (EGFR) and the involvement of this receptor in angiogenesis and metastasis processes, the inhibition of this receptor could offer a potential treatment for ovarian cancer [153, 154]. Macrophages that are activated by weakly induced apoptosis from the TNF family cytokine (TWEAK) [155] produce exosomes that contain some more microRNA-7 (miR-7) relative to macrophages that are activated commonly. These exosomes are captured by ovarian cancer cells, and miR-7 decreases these cells’ metastasis by inhibiting the EGFR/AKT/ERK1/2 signaling pathway [156, 157].

Due to the significant role of tumor-derived MSCs in angiogenesis and the invasion of tumor cells [158], the use of mechanisms that lead to the death of these cells reduces the rate of tumor angiogenesis and metastasis. Studies by Seo et al. reveal that exosomes produced by CD8^+^ T cells containing miR-298–5p reduce these cells’ population. Further, the study also showed that the transfer of CD8^+^ T cell exosomes produced by healthy mice could kill tumour-derived MSCs in the same species without H-2 (mouse MHC-1) restriction [159].

The results of new studies show that M1 macrophage-derived exosomes suppress proliferation, migration and invasion but lead to the induction of apoptosis in tumor cells. M1 exosomes affect tumor cell function by transferring lncRNA HOXA transcript at the distal tip (HOTTIP) to head and neck squamous cell carcinoma (HNSCC) [160]. However, the antitumor function is attenuated by HOTTIP-knockdown exosomes, indicating that HOTTIP acts as a key molecule in M1 exosomes. Further analysis of the role of HOTTIP lncRNA in tumor cells showed that overexpression of HOTTIP prevents proliferation, migration, and invasion but induces apoptosis of cancer cells in vitro [161]. Finally, the relevant data showed that exosomal lncRNA derived from M1 macrophages suppressed the progression of HNSCC by positively regulating the TLR5/NF-κB signaling pathway through competitive sponging miR-19a-3p and miR-19b-3p [161]. Also, it has been proven M1 macrophage-derived exosomes containing HOTTIP can induce circulating monocytes polarization to the M1 antitumor phenotype [161]. Therefore, it is suggested that the use of these exosomes may contribute to anti-tumor immunity. Also, Müller et al. Showed that NK cells derived exosomes could lead to the apoptosis of tumor cells. Further analysis showed that these exosomes inhibited the growth of MYCN- amplified neuroblastoma cells proliferation by transporting miR-186 [132, 162].

#### Decrease of drug resistance in tumor cells

Due to the inflammatory properties of M1 macrophages derived exosomes, they can be used in the treatment of various tumors. In a study by Zhang et al., cisplatin was loaded in exosomes (exoCIS) by sonication after separating from M1 macrophages. This study shows that the application of drug encapsulated exosomes leads to an increase in apoptosis of A2780/DDP (drug-resistant) and A2780 (drug-sensitive) cell lines compared to the use of cisplatin alone. Interestingly, cytotoxicity in drug-resistant cells has been significantly increased compared to sensitive cells. It seems that the two mechanisms are involved in the anti-cancer activity of exoCIS: (1) Preferred accumulation of cisplatin by exosome in cancer cells and (2) Intrinsic properties of exosomes derived from M1 macrophages to kill tumor cells [163].

### IEX therapeutic application in autoimmune diseases

Autoimmune diseases occur when abnormal immune system responses damage the body tissues and severely affect a patient’s quality of life. Due to immune system exosomes’ role in modulating immune responses, they are now used to treat many autoimmune diseases such as multiple sclerosis, rheumatoid arthritis, inflammatory bowel disease, and myasthenia gravis.

#### Improve multiple sclerosis (MS) and experimental autoimmune encephalomyelitis (EAE)

In vivo analysis show that the use of IFN-γ-induced DCs derived exosomes (IFN-γ-DEX) through the transfer of various miRNAs increases the central nervous system (CNS) myelination in patients with MS and demyelination syndromes. Among the miRs involved in this process is miR-219, which plays a crucial role in myelin formation and its maintenance [164], miR-9, which is produced during oligodendrocyte maturation [165], and increases the oligodendroglial differentiation, and miR-17–92 that increase the proliferation of oligodendrocyte progenitor cells [166]. Finally, it can be articulated that these exosomes stimulate CNS myelination by increasing the proliferation and differentiation of oligodendrocyte progenitor cells, increasing the antioxidant activity of microglia, and modulating the inflammatory environment [167].

Yu’s study showed that using membrane TGF-β1 expressing DEXs (mTGF-β1-EXOs) in mice with EAE inhibited the progression and development of the disease. Treatment of mice with these exosomes also reduced Ag-specific Th1 and IL-17 mediated responses. It increased IL-10 related responses in ex vivo as they help cure the disease by increasing Treg cells’ differentiation and reducing the inflammation-associated damages [168]. Given that T cell inflammatory responses are one of the important factors in the development of MS, the use of Treg derived exosomes to inhibit their function can be helpful; studies reveal that exosomes produced from Treg in people with MS have fewer immunomodulatory effects compared to healthy people. Therefore, exosomes derived from the Treg of healthy individuals can effectively treat autoimmune diseases such as MS and EAE. These exosomes reduce proliferation in conventional T cells and induce apoptosis to prevent T cell reactions [169].

#### Myasthenia gravity (MG)

A study by Bu et al. in 2015 showed that the use of immature dendritic cell exosomes (imDEX) prevented the progression of the experimental autoimmune Myasthenia Gravity (EAMG) in mice as they suppress the responses of B cells (producing anti-AchR antibody) and AchR specific T cells (producing inflammatory cytokines) [170]. In a study by Yin, miR-146a was used to produce tolerogenic DCs. These cells produce exosomes that express a reduced amount of CD80 and CD86 molecules, while the amount of MHC-II molecules on these exosomes’ surfaces has not changed. The results show that exosomes derived antigen-specific immature DCs reduce the clinical signs of EAMG mice by decreasing the serum levels of anti-AchR, IgG1, and IgG2b, as well as shifting the T cell responses from Th1/Th17 to Th2/Treg [171].

#### Inflammatory bowel disease (IBD)

Due to the anti-inflammatory properties of MDSC, they can be deployed in the treatment of autoimmune diseases [172], as illustrated in a study where this cell’s exosomes have been used to treat dextran sodium sulfate(DSS)-induced colitis. Due to the presence of high levels of arginase-1 (Arg-1) in MDSC-Exo, these exosomes suppress inflammatory responses and help treat induced colitis in mice by reducing the number of Th1 cells and increasing Treg. Also, it has been observed that serum levels of IFN-γ and TNF-α were decreased significantly in exosome-treated mice compared to controls. Interestingly, the inhibition of Arg-1 activity in MDSC-Exo significantly reduces the exosome therapy’s recovery effect [173].

#### Rheumatoid arthritis (RA)

RA is a chronic and systemic autoimmune disease in which chronic inflammation of the joints causes joint damage. The use of DEXs due to their characteristics is one way to reduce inflammation and treat this disease. Various studies show that some strategies have been applied to produce inflammation suppressing DCs (TolDCs) that produce such anti-inflammatory exosomes. These include genetically modified DCs for IL-4 expression [174], treatment of DCs with IL-10 [175], and enzyme indoleamine 2,3 deoxygenase (IDO) containing DCs [176]. These exosomes can modulate the APCs and T cells’ activity through the MHC-II- and FasL/Fas dependent mechanisms. Besides, they can also suppress delayed-type hypersensitivity (DTH)-related inflammatory responses and improve collagen-induced osteoarthritis [174]. Furthermore, various studies have shown that IDO-expressing DCs derived exosomes suppress the CD8^+^ T cells’ activity by reducing tryptophan dose and producing cytotoxic metabolites, leading to the enhanced differentiation and activation of Treg cells [176].

### IEX therapeutic application in transplant rejection

Some immune cells with tolerogenic and immunosuppressive properties produce exosomes that can be applied to prevent tissue transplant rejection in the host. For example, PD-L1 expressing TolDCs deliver the donor MHC molecules to the recipient by tolerogenic methods and suppress the response of effector T cells produced against transplantation through PD-L1 [177]. In a study by Yu et al., serum and histological analysis showed that using Treg CD4^+^ CD25^+^ exosomes leads to renal allografts’ long-term survival in the mouse model. Also, in vitro analyses revealed that the use of these exosomes suppresses T cell proliferation. Therefore, according to the results of in vivo and in vitro analyses, exosomes released from Tregs can be considered a potential immunosuppressive agent to prevent transplant rejection [178]. Furthermore, the exosomes produced by Tregs express inhibitory molecules such as CTLA-4 and CD25 on their surface [73]. After fusion of these exosomes with APCs, these molecules are placed on the surface of them and can inhibit the activation of T cells by removing IL-2 from the environment and reducing its amount by CD25 to inhibit the initiation of T cell responses by CTLA-4 [179].

### IEX therapeutic application in infectious diseases

IEXs play an important role in antimicrobial responses where these exosomes operate as “bridges” between different cells and prevent the spread of viral and bacterial infections. During hepatitis C virus (HCV) infection, the interaction between resident macrophages and liver cells is a critical component of the liver’s innate immunity. In 2016, Zhou et al. showed that the transfer of TLR-3 activated macrophages supernatants (that contain exosomes) to Huh7 cells (human liver cell line type) prevents the proliferation of HCV within them; these exosomes carry some members of the miRNA-29 family such as miR-29a, 29b, and 29c and have an anti-HCV role [180].

LPS activated mature CD8^+^ DCs secrete exosomes with high levels of MHC-1 and ICAM-1 at their surface, which are captured by other DCs through LFA-1-ICAM-1 interaction, and transfer the processed antigen and the peptide-MHC complex to the target cell. These DCs cannot reprocess antigens, but they can enhance T cell activation in the presence of exosome-associated MHC-peptide complex and the delivery of exogenous antigens. By this mechanism, activated DCs increase the number of APCs to T cells [181]. DEXs can also carry microbial TLR ligands and transfer them to other DCs to activate them and increase the antigen presentation and production of inflammatory cytokines such as TNF-α. Further, these exosomes increase NK cell function and differentiation of CD4^+^ T cells into Th1 [182]. Since more than 90% of healthy individuals produce effective responses against mycobacterium tuberculosis, and because infected DCs and macrophages have a limited ability to deliver antigens, other mechanisms such as exosome production play a very effective role in the activation of T cells [183]. The inhibition of exosome production by tuberculosis antigens presenting DCs and macrophages leads to decreased immune response and increased bacterial load. Defects in exosome production, such as those that occur in Rab27a-deficient mice, significantly reduce the bacterial antigen trafficking and immune system responses. So, DC and macrophage-derived exosomes can be used to stimulate T-cell mycobacterium tuberculosis-specific responses to and prevent the disease progression [184]. Another study that confirms the therapeutic function of IEXs in the treatment of microbial infections pertains to *Helicobacter pylori*-infected DCs and macrophages-derived exosomes. These exosomes carry miR-155 in large quantities and transport them to other macrophages during vesicular trafficking. Transfer of these exosomes to macrophages increases the production of inflammatory cytokines such as TNF-a, IL-6, IL-23, and costimulatory molecules such as CD40, CD63, CD81, and MHC-I [185, 186].

### IEX therapeutic application in asthma and allergies

Exosomes produced by mast cells contain receptors with a high affinity for IgE, and they can bind to free IgE in serum via the FC𝜀R1 and decrease the concentration of this antibody in the serum. This reduction in serum IgE limits the activation of other mast cells and prevents allergic reactions. The results of a study by Xie et al. reveal that the use of bone marrow-derived mast cell in mice with chronic asthma reduces respiratory tract inflammation and has a role in the remodelling and regeneration of lung tissue; accordingly, mast cell exosomes can be used as an anti-IgE agent in various allergic diseases [187, 188].

In 2020, Li et al. described that M2 macrophage-derived exosomes could prevent the progression of asthma. In this study, to create a model of asthma in C57BL/6 mice, they used ovalbumin (OVA) and Freund adjuvant. The outcome reveals that these exosomes carry a large amount of miR-370, inhibiting the FGF1/MAPK/STAT1 signaling axis in airway epithelial cells (AECs) [189]; they inhibit cell apoptosis, pulmonary fibrosis, OVA-induced inflammation in mice, and ectopic hyperplasia in AECs. Further studies illustrate the number of granulocytes, such as neutrophils and eosinophils, and the levels of IL-1β, IL-6, TNF-α, and MCP-1 in OVA-treated mice in bronchoalveolar lavage fluid (BALF) are reduced. These findings provide a new understanding of the involved molecules in asthma development and suggest the deployment of exosomes as an alternative treatment [190]. Due to the importance of IEXs clinical applications, Table 3 summarizes the cellular sources of exosomes, mediators, mechanism, type of disease, and their application results (Table 3).

**Table 3:** Summary for therapeutic applications of IEXs

Exosome source	Mediator	Target cell	Mediator target	Type of disease	Therapeutic outcomes
Dendritic cell (DC)	HSP70	Gastric cancer cell line	N/A	Gastric cancer	↑ Apoptosis and ↓ proliferation in gastric cancer cell line
M1 macrophages	miR-let-7a-5p	Lung cancer cells	BCL2-like protein 1 (BCL2-L1), insulin-like growth factor 1 receptor (IGF1R), mitogen-activated protein kinase 8 (MAPK8), and Fas	Lung cancer	↑ Autophagy and apoptosis in lung cancer cells
NK cell	DNAX-1 accessory molecule (DNAM1)	NALM-18 cell line	PVR and Nectin-2	Acute lymphoblastic leukaemia	1. Disrupts the tumor cell membranes integrity 2. ↑ Apoptosis in lung cancer cells
TWEAK activated macrophages	miR-7	Ovarian cancer cells	EGFR/AKT/ERK1/2 signaling pathway	ovarian cancer	↓ Metastasis
CD8^+^ T cells	miR-298–5p	Tumor-derived MSCs	cAMP/PKA-mediated manner	pancreatic cancer	1. Kill tumour-derived MSCs 2. ↓ Metastasis
M1 macrophage	HOTTIP	Tumor cells	TLR5/NF-κB signaling pathway	HNSCC	1. Prevents proliferation, migration, and invasion but induces apoptosis of cancer cells 2. Induce monocytes polarization to the M1 antitumor phenotype
NK cell	miR-186	Tumor cells	TGF-β1-dependent manner	Neuroblastoma	1. ↑ Apoptosis and ↓ proliferation in tumor cell 2. ↓ Immune escape in high-risk neuroblastoma patients
IFN-γ-induced DCs	miR-219, miR-9, miR-17–92	Oligodendrocyte, brain resident cells	N/A	Multiple Sclerosis	1. ↑ proliferation and differentiation of oligodendrocyte progenitor cells 2. ↑ Antioxidant activity of microglia 3. Modulating the inflammatory environment
Immature DCs	N/A	B lymphocytes, T lymphocytes,	Suppress the anti-AchR antibody-producing B cells and AchR specific T cells responses	Myasthenia Gravity	1. ↓ Serum levels of anti-AchR, IgG1, and IgG2b 2. Shift T cell responses from Th1/Th17 to Th2/Treg
Myeloid-derived suppressor cells (MDSCs)	Arginase-1 (Arg-1)	TCD4^+^ cells	Conversion of arginine to ornithine and urea	Dextran sodium sulfate (DSS)-induced colitis	1. ↓ the number of Th1 cells 2. ↑ the number of Treg cells 3. ↓ IFN-γ and TNF-α level in serum
Regulatory T cells	CTLA-4 and CD25	TCD4^+^ cells	Suppress T cell activation (by CTLA-4), removing IL-2 from the environment (by CD25)	Tissue transplant	1. ↑ Renal allografts’ long-term survival 2. Suppresses T cell proliferation
M1 macrophage	miR-29a, 29b, and 29c	Huh7 cells	N/A	Hepatitis C virus (HCV) infection	↑ Anti-HCV immunity
LPS activated mature CD8^+^ DCs	MHC-1 and ICAM-1, microbial TLR ligands	DCs	LFA-1-ICAM-1 interaction, transfer the processed antigen and the peptide-MHC complex	Infectious diseases	1. ↑ Number of activated DCs 2. ↑ Production of inflammatory cytokines 3. ↑ NK cell function 4. ↑ Differentiation of CD4+ T cells into Th1
DCs and macrophages	miR-155	Macrophages	NF-κB signaling pathway	*Helicobacter pylori* infection	1. ↑ Inflammatory cytokines such as TNF-a, IL-6, IL-23 2. ↑ Costimulatory molecules such as CD40, CD63, CD81, and MHC-I
Mast cells	FC𝜀R1	B lymphocyte	Free IgE	Asthma and allergies	↓ Concentration of IgE
M2 macrophage	miR-370	Airway epithelial cells (AECs)	FGF1/MAPK/STAT1 signaling axis	Asthma	1. ↓ Cell apoptosis 2. ↓ Pulmonary fibrosis 3. ↓ OVA-induced inflammation in mice and ectopic hyperplasia in AECs 4. ↓ Number of granulocytes, such as neutrophils and eosinophils 5. ↓ Levels of IL-1β, IL-6, TNF-α, and MCP-1

*ICAM-1* Intercellular adhesion molecule 1, *APAF1* Apoptotic peptidase activating factor 1, *PTEN* Phosphatase and tensin homolog, *NF-κB* Nuclear factor-kappa B, *NK cell* Natural killer cells, *TNF-α* Tumor necrosis factor-alpha, *IL* Interleukin, *MCP-1* Monocyte Chemoattractant Protein-1, *OVA* Ovalbumin, *MHC* Major histocompatibility complex, *TLR* Toll-like receptor, *AchR* Acetylcholine receptor, *TGF-β1* Transforming growth factor-beta 1, *N/A* Not applicable

## Conclusions and future prospects

As shown in Table 4, despite all the advantages of IEXs, only a handful of clinical trials have exploited these nanovesicles. Due to the existence of some unresolved limitations, the clinical application of exosomes has been challenging [191]. For example, the biological distribution of these vesicles in the individuals treated by them is not well understood, which may lead to difficult challenges in managing the use of exosomes in standardization methods related to their separation, quantification, and analysis of results [83]. Also, the difficulty in exosome storage due to their aggregation and the destruction of their cargo during the freeze-thaw process is another limitation of their limitations [192]. This problem can be circumvented by adding stabilizers such as sucrose, trehalose, and glucose [193]. The choice of an appropriate source for exosome isolation in therapeutic applications and as a delivery system for each molecule and drug is a significant issue that is often completely ignored and can greatly affect treatment outcomes [4].

**Table 4:** IEXs application in clinical trials

NCT Numbers	Exosome Source	Country	Status	Started in
NCT02957279	Dendritic cell	China	Recruiting	2016
NCT01159288	Dendritic cell	France	Completed	2018
NCT04389385	T cell	turkey	Active	2020

Moreover, the clinical trials using IEXs show that naive exosomes are often insufficient to produce strong responses in vivo [194]. The focus has now shifted to improving the effects of exosomes by engineering them for different treatment approaches. One of these strategies is to load drugs in IEXs [195]. An example of this type of exosome is the treatment of tumors using doxorubicin encapsulated DEXs [196].

The interaction between immune system cells through the exosomes is very complex. Exosome production by immune cells leads to change in the function of adjacent cells (paracrine effect), and sometimes the cells themselves (autocrine effect), and turn them into better cells to perform their functions. Besides, the IEXs can be responsible for tissue damage caused by many diseases, such as asthma. Therefore, the function of these exosomes is not always beneficial to the body and sometimes disrupts the function of the other cells. However, due to the unique features of exosomes, including biocompatibility, low clearance rate, and ability to transfer drugs, they can be used as drug delivery vehicles [132]. Understanding the properties of diverse cell exosomes is crucial for selecting an appropriate exosome for therapeutic applications. Most of IEXs can induce inflammation, and hence they can be used to enhance immune responses to infections and tumors. In contrast, some IEXs have inhibitory roles on immune system responses that can be used to prevent graft rejection and treatment of hypersensitivity and autoimmune diseases.

Finally, it can be said that studies in this field are in their beginning, and more research is needed on the wide therapeutic application of IEX in the treatment of various diseases.

## Data Availability

The data supporting the conclusions of this article are all online.

## Abbreviations

IEXs: Immune cells-derived exosomes
DEXs: Dendritic cell-derived exosomes
APCs: Antigen-presenting cells
MVBs: Multivesicular bodies
ILVs: Intraluminal vesicles
ESCRT: Endosomal sorting complex required for transport
MHC: Major histocompatibility complex
HSPs: Heat shock proteins
DCs: Dendritic cells
FAS-L: Fas ligand
LPS: Lipopolysaccharide
TLR: TOLL-like receptor
TNF-a: Tumour necrosis factor-alpha
PI3-k: Phosphoinositide 3-kinase
FDCs: Follicular dendritic cells
ECM: Extracellular matrix
TCR: T cell receptor
MMP: Matrix metalloproteinase
PTEN: Phosphatase and tensin homolog
lncRNA: Long non-coding RNA
HIF-1a: Hypoxia-inducible factor 1-alpha
